# Supplementary material for: Seroprevalence of Equine Influenza Virus Antibodies in Horses from Four Localities in Colombia
Source: Viruses. 2025 Jul 16;17(7):999. doi: 10.3390/v17070999 (PMC12299346; doi:10.3390/v17070999)
Supplement: Supplementary file 1 [file viruses-17-00999-s001.zip › viruses-3698481-supplementary.pdf]

Supplementary table S1: Questionnaire Study 1

Equine data collection form	
**Horse ID/Name:**	
**Breed:**	
**Sex:**	
**Age (years):**	
**Locality:**	
**Type of shelter:**	
**Type of Activity:**	
**Influenza Vaccination:**	
**Clinical Signs**	
**Bilateral Nasa Discharge**	Yes __ No
**Cough**	Yes__No
**Fever**	Yes__No
**Respiratory distress**	Yes__No

Supplementary table S2: Questionnaire Study 2

Equine data collection form
**Horse ID/Name:**
**Breed:**
**Sex:**
**Age (years):**
**Locality:**
**Type of shelter:**
**Type of Activity:**

https://doi.org/10.3390/xxxxx
